# Arrangement optimization of water-driven triboelectric nanogenerators considering capillary phenomenon between hydrophobic surfaces

**DOI:** 10.1038/s41598-020-57851-9

**Published:** 2020-01-24

**Authors:** Hong Ryul Park, Jeong-Won Lee, Dong Sung Kim, Jae-Yoon Sim, Insang Song, Woonbong Hwang

**Affiliations:** 1 Department of Mechanical Engineering, Pohang University of Science and Technology (POSTECH), Pohang, 37673 Korea; 2 Department of Electrical Engineering, Pohang University of Science and Technology (POSTECH), Pohang, 37673 Korea; 3 Agency for Defense Development (ADD), Daejeon, 34186 Korea

**Keywords:** Energy science and technology, Materials science, Nanoscience and technology

## Abstract

The rise in environmental issues has stimulated research on alternative energy. In this regard, triboelectric generation has received much attention as one of several new alternative energy sources. Among the triboelectric generation methods, solid-liquid triboelectric nanogenerators (SLTENGs) have been actively investigated owing to their durability and broad applicability. In this paper, we report on the optimum arrangement of SLTENGs to increase the generation of electrical energy. When hydrophobic SLTENGs are arranged in parallel with a specific intervening gap, the friction area between the water and the surface of the SLTENGs is changed owing to the different penetration distances of water between them. This difference affects the amount of triboelectricity generated; this change in the water contact area is caused by the capillary phenomenon. Therefore, we investigated the effect of the gap on water penetration and formulated an optimum arrangement to achieve optimum electricity generation efficiency when multiple SLTENGs are contained in a limited volume. The proposed optimum arrangement of SLTENGs is expected to have high utilization in energy harvesting from natural environment sources such as wave energy or water flow.

## Introduction

Nowadays, environmental issues such as global warming and climate change have become severe owing to the indiscriminate use of fossil energy. Moreover, the price of fossil energy will increase steadily because it is a finite resource. Therefore, many researchers have endeavoured to develop alternative green energies using sunlight, wind, tide, geothermal heat, and the like^1–10^. Among alternative energies, triboelectric nanogenerator (TENG) has received much attention in academia. TENG is a new invention that harvests energy using triboelectricity, which is common in our lives. Triboelectrification, also called contact electrification, is a common phenomenon that occurs when two different materials are in contact with each other^11–33^. Triboelectrification is commonly classified as solid-solid contact electrification and solid-liquid contact electrification^34,35^. Solid-solid contact electrification generally generates more energy than the solid-liquid type, but it is difficult to obtain sustainable energy from this owing to surface abrasion. In other words, the solid-liquid contact type can generate lower but continuous energy because the contact between solid and liquid does not cause abrasion of the solid surface. Therefore, studies have been carried out to amplify the amount of energy output through modification of the surface structure or stacking multiple TENGs^36–38^. Lee *et al*. reported on the effect of surface roughness on solid-liquid triboelectrification and proposed a method for fabricating high-performance solid-liquid type TENG^39–45^. When hydrophobic TENGs are stacked in parallel and submerged into a liquid, the penetration distance of the liquid into the gap between the TENGs varies owing to the capillary effect. Consequently, the effect leads to difference in the electricity generation.

In this study, we developed an arrangement of TENGs that allows the most efficient energy output within a limited volume by considering the capillary effect. First, the gap between parallel-stacked solid-liquid TENGs (SLTENGs) that generates the maximum electrical output was investigated. Next, we analysed whether this optimal value of the gap is effective when the number of electrodes is varied within the limited volume. Through experimentation, the optimal arrangement of SLTENGs in a limited volume was determined for sustainable and increased electricity generation. The proposed arrangement can be regarded as a guide for increasing the electrical output of liquid-driven TENGs.

## Experimental Section

### Materials

Industrial aluminium plates (Al 5052, thickness: 1.0 mm) were purchased from Alfa Aesar, USA. Perchloric acid (HClO_4_), ethyl alcohol (C_2_H_5_OH), oxalic acid (C_2_H_2_O_4_), and n-hexane (C_6_H_14_) were obtained from SAMCHUN Chemicals, Republic of Korea. Heptadecafluoro-1, 1, 2, 2-tetrahydrodecyl trichlorosilane (HDFS) was supplied by JSI Silicone Co., Japan. Polytetrafluoroethylene (PTFE) was purchased from Du Pont, USA. All other chemicals were of analytical reagent grade and were used as received.

### Fabrication and characterization

The proposed SLTENG was fabricated as follows. To eliminate the impurities and unevenness that influence contact electrification on industrial aluminium plates, the plates were polished in a mixture of perchloric acid and ethyl alcohol (HClO_4_: C_2_H_5_OH = 1: 4 by volume) under a constant voltage of 20 V for 5 min using a computer power supply (Digital Electronics Co., DRP-92001DUS). The mixture was maintained at 7 °C during the polishing process by means of a circulator (Lab Companion, RW-0525G). After the polishing process, the plates were rinsed with deionized (DI) water and dried. To produce the anodized aluminium oxide (AAO) layer, which serves as a dielectric layer to hold the charge by triboelectrification, the as-prepared aluminium plates were anodized in 0.3 M oxalic acid solution maintained at 25 °C under a constant voltage of 50 V for 20 min using the computer power supply^39,46^. After the anodizing process, the plates were rinsed with DI water and dried. The anodized aluminium plates were dipped in a mixture of n-hexane and HDFS with volumetric ratio of 1000:1 for 10 min, followed by drying at 60 °C for 6 h. Then, PTFE was added to the surface to form an over-coating of the PTFE layer to cause the surface to become negatively charged in the triboelectric series. The plates were then washed with DI water and dried in a stream of air.

A field emission scanning electron microscope (FESEM; JEOL JSM-7401F, Japan) was used to observe the anodized Al surface and to measure the thickness of the anodized layer. The contact angle (CA) was measured with 5-μl water droplets using a CA measurement system (SmartDrop_Lab HS, FEMTOFAB, Republic of Korea). The average CA value was obtained by measuring each sample at a minimum of three different positions at room temperature. The optical images of the droplets were obtained using a digital camera.

### Electrical output measurement

The fabricated SLTENGs were vertically mounted on an electrodynamic shaker (LABWORKS) and another electrode was attached to a beaker and placed below the shaker. The beaker was filled with 600 ml of DI water. The SLTENGs were then repeatedly dipped in water at a frequency of 2 Hz and dipping depth of 1 cm. The energy generated from water-solid contact electrification was analysed by measuring the output current and output voltage under the load resistance of 10 MΩ using an oscilloscope (DS1000Z, Rigol) and low-noise current pre-amplifier (SR570, Stanford Research Systems)^39^.

## Results and Discussion

### Water-solid contact electrification of SLTENG

When the sliding contact electrification occurs, the charge and voltage follow the formula:

If the immersed depth is defined as *x*, the total charge (*Q*) is given by

1 $$Q=\sigma \times {A}_{contact}=\sigma wx,$$

where *σ* is the surface charge density and *w* is the width of the SLTENG. With regard to the relative interfacial velocity (*v*_*r*_(*t*)), the output current (*I*) can be expressed as

2 $$I=\frac{dQ}{dt}=\sigma w\frac{dx}{dt}=\sigma w{v}_{r}(t)$$

The output voltage (*V*) can be expressed as

3 $$V=\frac{{\rm{\sigma }}dx}{{\varepsilon }_{0}{\varepsilon }_{r}(l-x)},$$

where *d* and *l* are the effective dielectric thickness and the total friction length, respectively; *ε*_0_ and *ε*_*r*_ are the dielectric constant of vacuum and relative permittivity of the dielectric material, respectively^46,47^.

**Figure 1:**
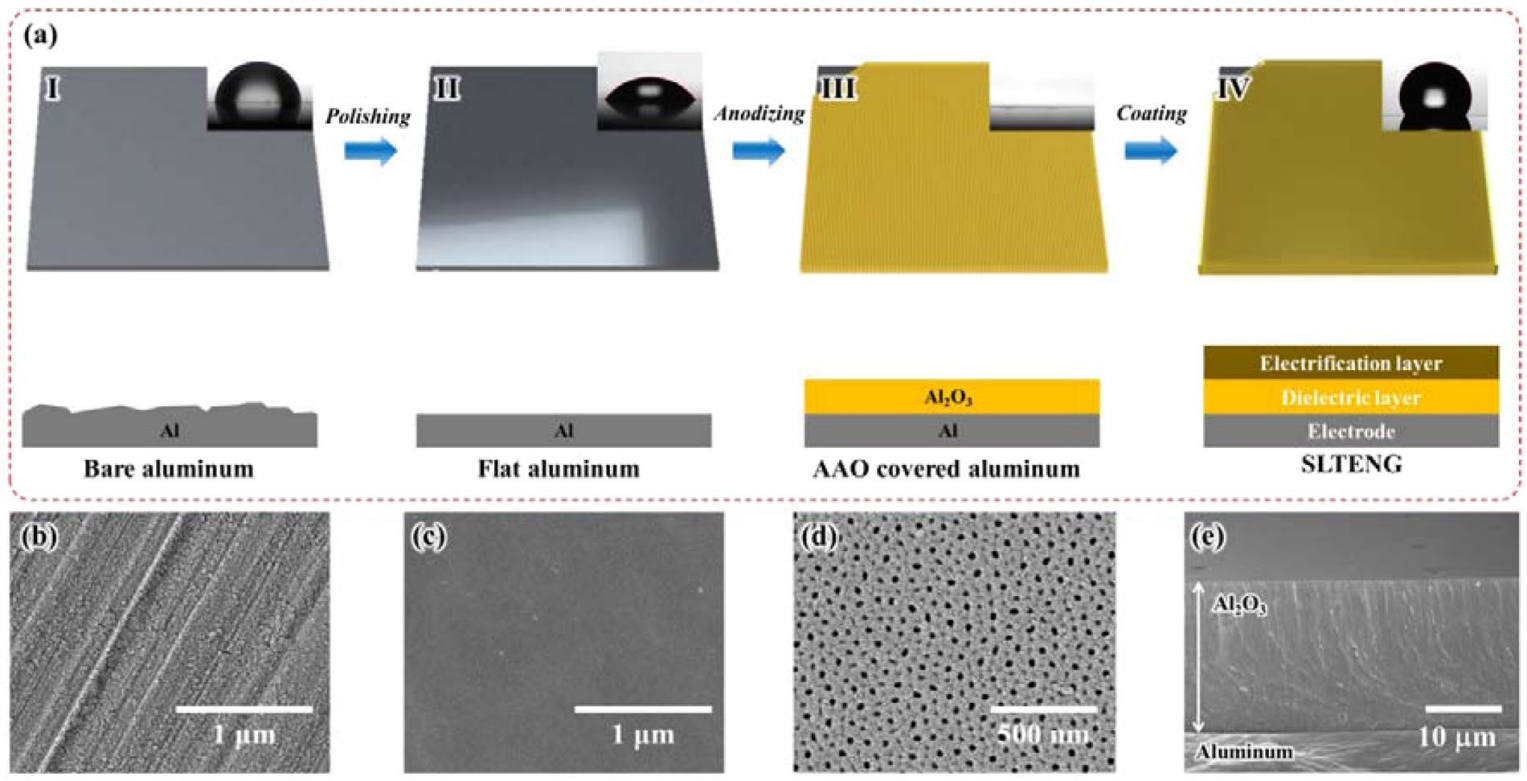
(**a**) Fabrication process of the solid-liquid triboelectric nanogenerator (SLTENG) with contact angles (CAs). Scanning electron microscopy (SEM) images of (**b**) bare aluminum, (**c**) electropolished aluminum, (**d**) anodized aluminum. (**e**) Cross-sectional SEM image of Al_2_O_3_.

In triboelectric energy generation, definite contact and separation, and large contact area between liquid and the solid surface are critical factors for high electrical output. Therefore, a hydrophobic nano-hole structured surface of SLTENG was fabricated, as shown in Fig. 1(a). The surface roughness and impurities of bare aluminium were flattened and cleaned by electropolishing (Fig. 1(b,c)). The removal of surface impurities slightly enhanced the hydrophilicity (Fig. 1(a-I,a-II)). Through the subsequent anodization process, a superhydrophilic Al_2_O_3_ surface with evenly distributed 20-nm nanoholes was fabricated (Fig. 1(a-III,d)). Both the HDFS and the PTFE self-assembled monolayer (SAM) coatings chemically modify the surface and make it hydrophobic without making any changes to the surface structures. Although the SAM coating of HDFS can solely function as a hydrophobic surface, the chain formation of PTFE, –(CF_2_–CF_2_)_n_–, increases durability against abrasion caused by continuous sliding friction of water and the surface.

The fabricated SLTENG comprised three layers: electrode, dielectric layer, and electrification layer (Fig. 1(a-IV)). The dielectric layer was the AAO layer of approximately 20-μm thickness, which provided insulation between liquid and the electrode (Fig. 1(e)). The electrification layer, which loses or gains electrons on contact, consisted of a PTFE coating. PTFE tends to be negatively charged in a triboelectric series. The SLTENG had a CA of 120°.

**Figure 2:**
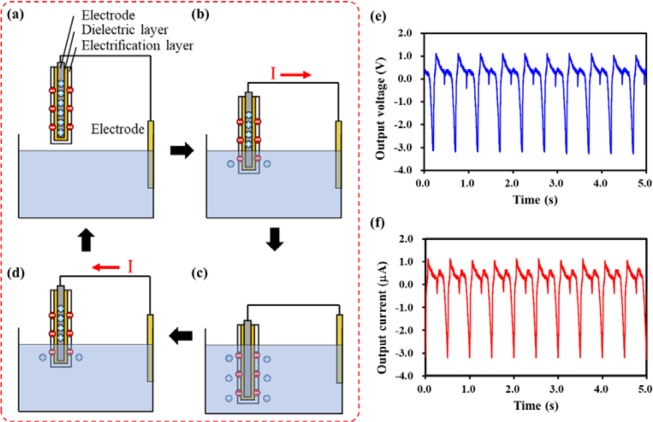
(**a**–**d**) Schematic of solid-liquid triboelectrification process of SLTENG. (**e**) Output voltage and (**f**) output current of single SLTENG.

The simplified solid-liquid triboelectrification process of SLTENG is shown in Fig. 2. Before the SLTENG contacts with water, the entire system, including the SLTENG and water, is in a state of electrical equilibrium (Fig. 2(a)). When the SLTENG comes into contact with water, the electrification layer of the SLTENG and water are respectively electrified negatively and positively due to the triboelectric series and current flows from the SLTENG to water to maintain the electrical equilibrium (Fig. 2(b)). When fully dipped in water, the SLTENG and water are in electrical equilibrium (Fig. 2(c)). When the SLTENG is out of the water, the surface of the SLTENG and water are electrified and current flows reversely (Fig. 2(d)). When the SLTENG is fully out of water, the state is the same as in Fig. 2(a) and the process is repeated. The electrical output measurements of a single SLTENG are shown in Fig. 2(e,f). The output voltage and current of a single SLTENG are approximately 3 V and 3.2 μA, respectively, at a frequency of 2 Hz and amplitude of 1 cm using the electrodynamic shaker. The power variation by frequency and amplitude is shown in Fig. S1. The amount of electricity generated differs according to the frequency and amplitude, but we applied 2 Hz and 1 cm as the natural wave environment in this experiment. A parallel arrangement with a specific gap was devised to increase the amount of electricity generation.

### Gap analysis between SLTENGs considering capillary effect

**Figure 3:**
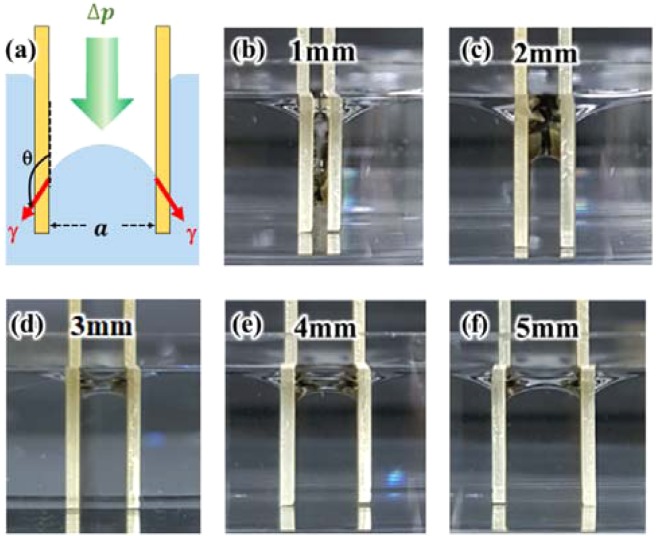
(**a**) Formation of capillary pressure between SLTENGs. (**b**–**f**) Optical images of water penetration with change in gap size from 1 mm to 5 mm.

An experimental model was devised to analyse the amount of electrical output based on the size of the gap between the SLTENGs arranged in parallel. Increasing in the number of electrodes resulted in increased electricity generation, but the volume of the entire TENG system also increased. In order to reduce the volume, it is essential to apply a narrower gap when multiple SLTENGs are stacked. However, the narrow gap increases the capillary pressure, which resists the penetration of water into the gap between the SLTENGs because of their hydrophobic property (Fig. 3(a)). Thus, the surface contact area with the water decreases and this leads to decrease in the electrical output. Capillary pressure is the difference in the pressures at the interface between two immiscible fluids such as water and air (Eq. (4)).

The capillary pressure Δ*p* is expressed as^48^,

4 $$\Delta p=\frac{2\gamma cos\theta }{a},$$

where *a*, *γ*, and *θ* are the radius of the capillary, the liquid/vapor interfacial tension, and the contact angle of the material, respectively. As the gap decreases, the capillary pressure increases. In the case of a hydrophobic surface, the pressure is negative, which prevents water from penetrating the capillary. In this experiment, the gap is the sole parameter because the applied surfaces have the same wetting properties. As the gap between the hydrophobic plates varies, the height of the water in the gap varies owing to negative capillary pressure.

To identify the quantity of water that penetrates between the hydrophobic plates, stacked SLTENGs separated by a specific gap were immersed in a circular chalet with 1-cm-deep water. The heights to which water had penetrated between the plates were observed while increasing the gap between two surfaces from 1 mm to 5 mm (Fig. 3(b–f)). As shown in the figure, the equilibrium of force is reached at the lower water level between the plates than outside the plates because of the higher capillary pressure. The penetration height of water between the plates is the lowest at the gap of 1 mm, which means that the capillary pressure is the maximum at 1 mm. As the gap increases gradually, the water level also increases. After 3 mm, it becomes almost equal to the height of the water outside the plates. This is because the influence of the capillary pressure generated between the plates is negligible, irrespective of the gap. Computer Aided Engineering (CAE) was performed to verify the height of water penetration between SLTENGs due to capillary phenomenon. A commercial program, ANSYS Fluent, was used for the analysis, and the result showed the same tendency of water penetration (Fig. S2). Also, the same tendency was observed under the real-time condition (Fig. S3).

#### Gap optimization between two SLTENGs

**Figure 4:**
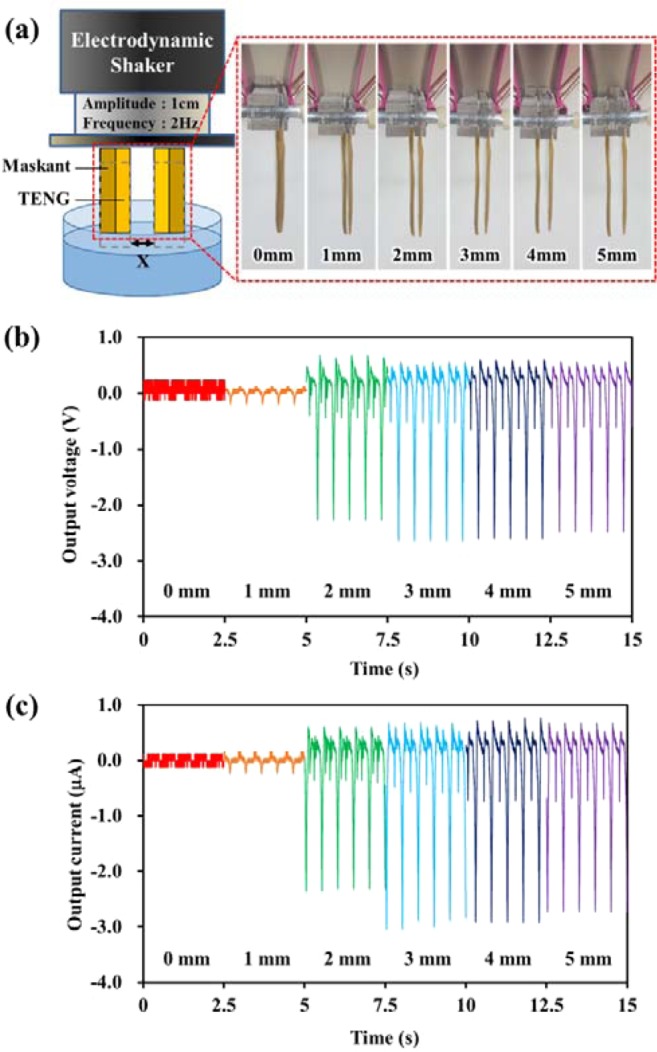
(**a**) Experimental setup for gap optimization. (**b**) Output voltage and (**c**) output current by altering the gap between two SLTENGs.

Figure 4(a) shows the actual experimental setup and illustration of the gap optimization between two SLTENGs. To investigate the change in electrical generation with gap between two SLTENGs, the sides and back of the fabricated SLTENGs were insulated using a maskant. The insulation was applied to obtain the electricity generated only from the inner gap between the two SLTENGs. The gap between the two SLTENGs was varied from 0 mm to 5 mm and their electrical energy evaluated at a frequency of 2 Hz and amplitude of 1 cm using the electrodynamic shaker.

The results are shown in Fig. 4(b,c). When the gap between the SLTENGs was 0 mm, the electrical output was virtually zero because there was no contact with water. When the gap was 1 mm, the measured generation value was 0.2 V and 0.25 μA. In spite of the 1-mm gap between the SLTENGs, the power generation was very low. Because the capillary pressure, which is inversely proportional to the gap, interfered with the penetration of water, only a small contact area existed between the SLTENG surface and water. In other words, the electrical output increased with the gap size because of the lowering of the capillary pressure. As a result, the power output gradually increased as the gap increased from 0 mm to 3 mm, and the maximum electrical output was 2.6 V and 3 μA at a gap of 3 mm. This amount was slightly lower than the energy output of the single SLTENG because the sides and back of the SLTENGs were insulated by the maskant. The measured energies at gaps of 4 mm and 5 mm were almost the same as that at the 3-mm gap. It is considered that the capillary pressure no longer affects water penetration when the gap increases beyond 3 mm, as observed in the previous gap analysis experiment. Therefore, the 3-mm gap between two SLTENGs is the best gap for maximum electrical output.

#### Areal density optimization in limited volume

**Figure 5:**
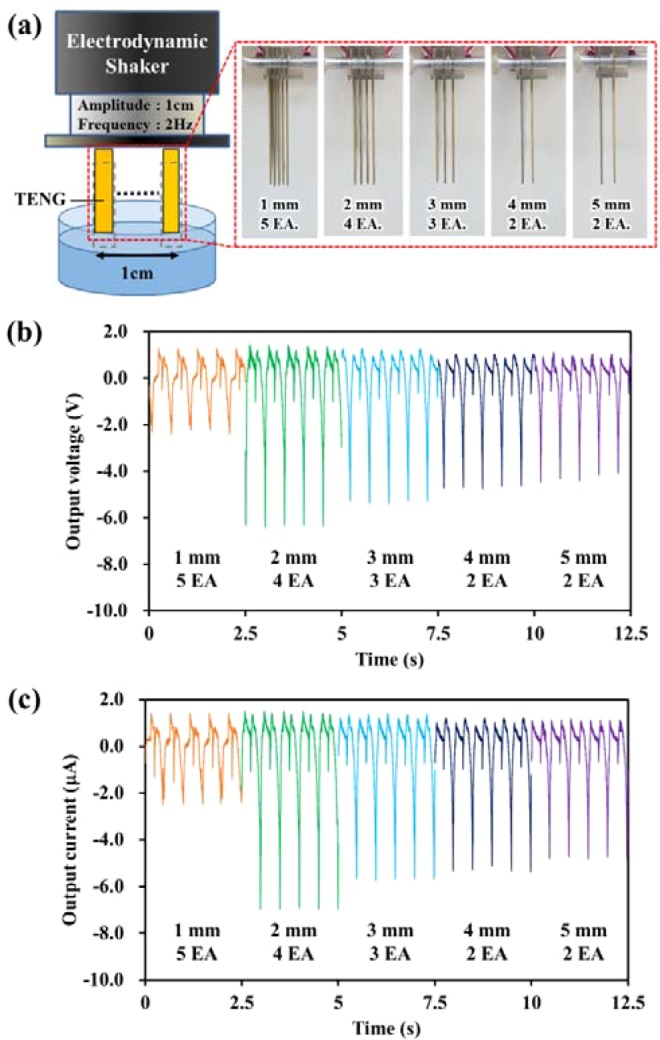
(**a**) Experimental setup for areal density optimization. (**b**) Output voltage and (**c**) output current by altering the gap between the SLTENGs within a limited volume.

The number of electrodes is also a critical factor for arranging electrodes efficiently with a specific gap within a limited volume. Therefore, it is necessary to study the dependence of power generation on the number of electrodes and the gap between the electrodes in a limited volume. Consequently, five electrodes with a 1-mm gap, four electrodes with a 2-mm gap, three electrodes with a 3-mm gap, and two pairs of electrodes with 4-mm and 5-mm gaps were placed within a limited distance of 1 cm (Fig. 5(a)). Other conditions for measurement were the same as in the gap optimization experiment.

The electrical outputs depending on the number of electrodes and the gap are shown in Fig. 5(b,c). Both output voltage and current were measured by a probe with an impedance of 10 MΩ. For reference, high-impedance probe measurement for open-circuit voltage was conducted and only the water penetration height was affected regardless of the contact area (Fig. S4). As shown in Fig. 5, the electrical outputs of the five electrodes with 1-mm gap are the lowest because the contact area of the SLTENGs with water is the smallest, as also shown in the gap optimization test. However, unlike in the previous test, the highest power occurs at the 2-mm gap. The power density can be expressed in watts, the product of voltage and current. Therefore, power density also follows the same trends of voltage and current, showing the highest amount of energy at 2 mm-gap. The highest power density was 8.84 μW/cm^3^, which was over 7 times higher than the same volume of 1.15 μW/cm^3^ with 1 mm-gap condition. This result shows that the number of electrodes has greater influence on the power generation than electrode spacing when water in the gap penetrates to a certain height in the limited volume. Therefore, for efficient enhancement of triboelectric power generation, it can be concluded that while increasing the number of SLTENGs, due consideration should be given to the limited volume and the capillary phenomenon.

## Conclusions

In this paper, we proposed an optimum arrangement for SLTENGs for improving the power generation from solid-liquid triboelectrification. When multiple SLTENGs are arranged to increase the contact area with the liquid, a narrow gap between the SLTENGs needs to be avoided to allow water to penetrate into the gap. On the other hand, the gap size should not be excessively large to ensure high areal density considering the limited volume. Therefore, the optimum gap size for the highest efficiency of the SLTENGs was experimentally investigated. While 3 mm was the optimum gap size when considering solely the capillary phenomenon, the maximum power was generated at 2 mm-gap size considering the limited volume. Furthermore, although aluminium-based TENGs were used in this study, the applications of this study are not limited to the materials or the surface property. Rather, it can be applied to a variety of applications using water flow or wave power to generate a continuous electrical output, which is an advantage of solid-liquid TENG.

## Supplementary information

Supplementary Information.
