# Supplementary material for: Arrangement optimization of water-driven triboelectric nanogenerators considering capillary phenomenon between hydrophobic surfaces
Source: Sci Rep. 2020 Jan 24;10:1126. doi: 10.1038/s41598-020-57851-9 (PMC6981242; doi:10.1038/s41598-020-57851-9)
Supplement: Supplementary file 1 — Supplementary Information. [file 41598_2020_57851_MOESM1_ESM.docx]

**Supplementary Information**

**Arrangement optimization of water-driven triboelectric nanogenerators considering capillary phenomenon between hydrophobic surfaces**

*Hong Ryul Park**^1,#^, Jeong-Won Lee^1,#^, Dong Sung Kim^1^, Jae-Yoon Sim^2^, Insang Song^3^ and Woonbong Hwang^1,*^*

# These authors contributed equally to this work as first authors

*E-mail: [whwang@postech.ac.kr](mailto:whwang@postech.ac.kr)

^1^Department of Mechanical Engineering, Pohang University of Science and Technology (POSTECH), Pohang, 37673, Republic of Korea

^2^Department of Electrical Engineering, Pohang University of Science and Technology (POSTECH), Pohang 37673, Korea

^3^Agency for Defense Development (ADD), Daejeon 34186, Korea


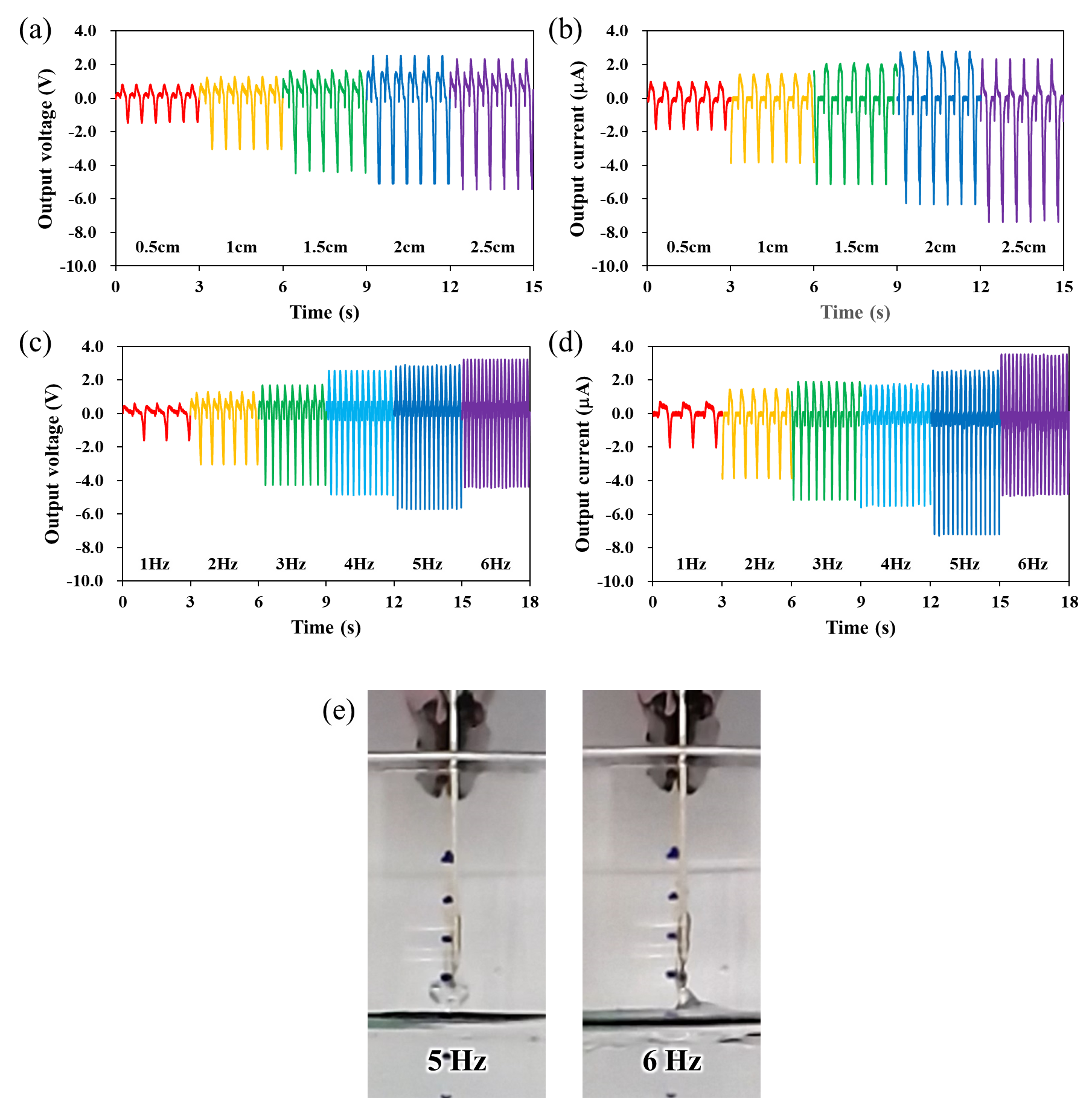


Figure S1. (a) Output voltage and (b) output current of single SLTENG for dipping depths ranging from 0.5 cm to 2.5 cm. (c) Output voltage and (d) output current of single SLTENG for frequencies ranging from 1 Hz to 6 Hz. (e) Optical images of water separation at frequencies of 5 Hz and 6 Hz

Description of Fig. S1:

Fig. S1a, S1b are graphs showing the relationship between the dipping depth and the amount of power for a single SLTENG. As shown in the above graph, the amount of power increases proportionally with the dipping depth. This is due to the increased contact area between water and electrode as the dipping depth increases. Fig. S1c, S1d are graphs showing the relationship between the frequency and power of a single SLTENG. As the frequency increases from 1 to 5 Hz, the power generation increases. However, the power generation decreases at 6 Hz. As shown in Fig. S1e, water is completely separated at 5 Hz, but a “water column” shape is observed at 6 Hz owing to insufficient separation of water. This is why the amount of electrical power is reduced at 6 Hz.


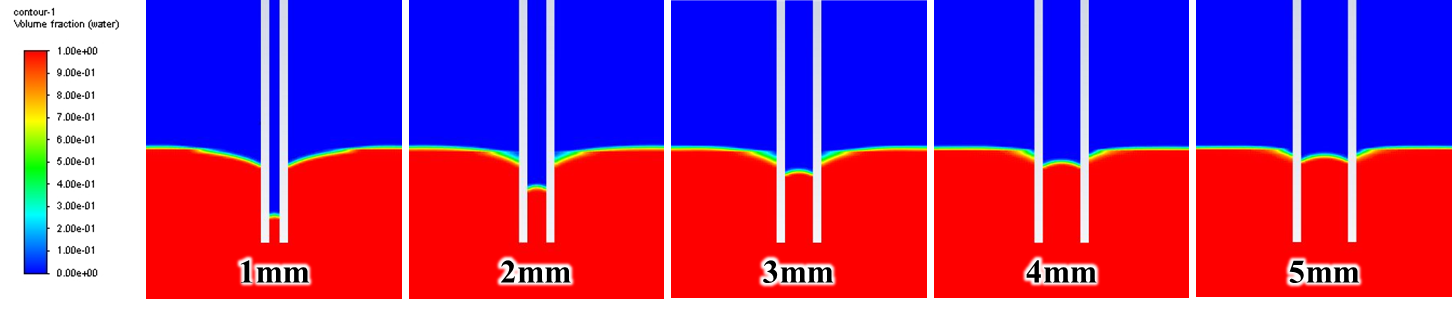


Figure S2. Theoretical simulation result of Computer Aided Engineering (CAE). Images shows the calculated water penetration height as the interval of SLTENGs changes to 1 ~ 5 mm.

Description of Fig. S2:

A commercial program ANSYS Fluent was used for the analysis, and the model was developed in 2D by simplifying the actual experimental model. The water contact angle of SLTENG was set to about 120° in the same way as the actual conditions, and it was designed to be immersed in water by 1cm with changing the interval of SLTENGs to 1 ~ 5 mm. The visualization of the analysis results was set to display the volume fraction of water as a contour. Water was set to red (volume fraction of water value: 1) and air to blue (volume fraction of water value: 0). As a result, as shown in the figure below, as the gap increases, the penetration height of the water gradually increases, so that it is saturated to a level similar to that of the outside water level of SLTENG.


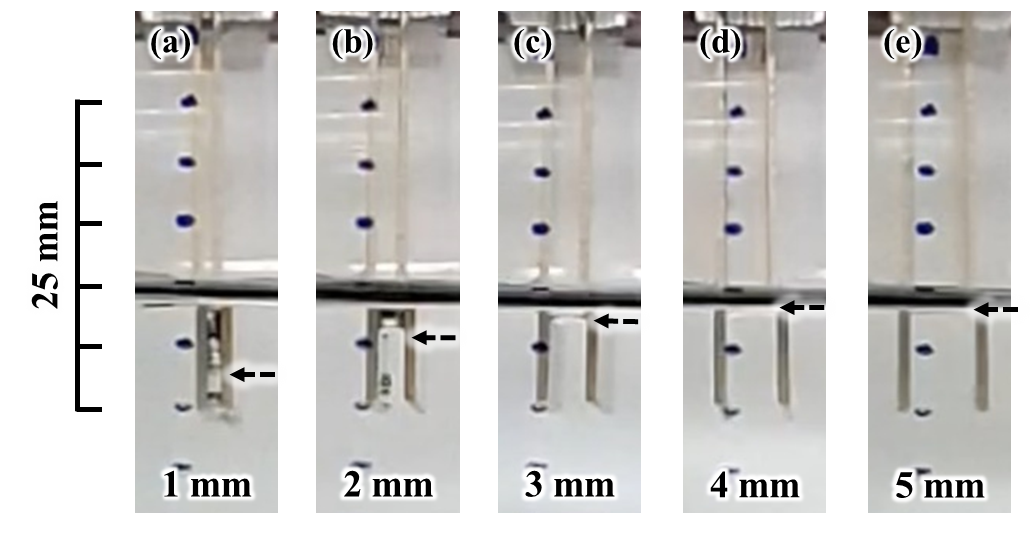


Figure S3. (a-e) Optical images of water penetration with change in gap size from 1 mm to 5 mm at a frequency of 2 Hz and amplitude of 1 cm using the electrodynamic shaker


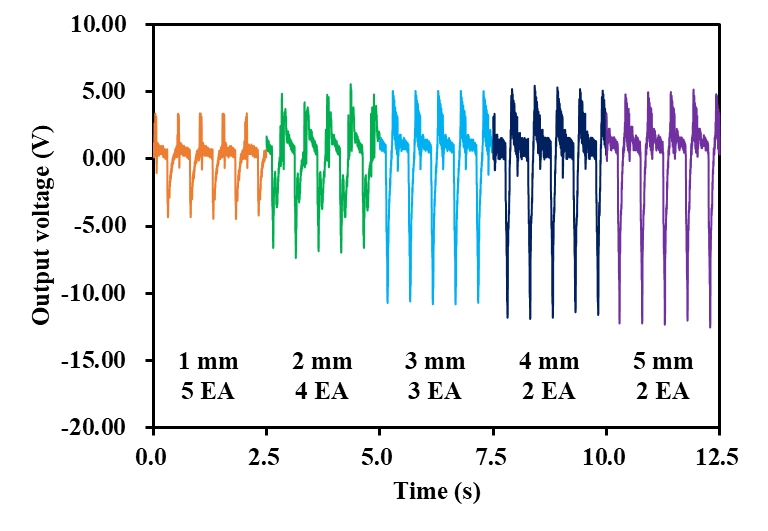


Figure S4. Output voltage at high-impedance by altering the gap between the SLTENGs within a limited volume.
